# Investigating the impact of pre-processing techniques and pre-trained word embeddings in detecting Arabic health information on social media

**DOI:** 10.1186/s40537-021-00488-w

**Published:** 2021-07-02

**Authors:** Yahya Albalawi, Jim Buckley, Nikola S. Nikolov

**Affiliations:** 1 Department of Computer Science and Information Systems, University of Limerick, Limerick, Ireland; 2 Department of Computer and Information Sciences, College of Arts and Science, University of Taibah, Al-Ula, Saudi Arabia; 3 The Irish Software Research Centre, Lero, University of Limerick, Limerick, Ireland

**Keywords:** Deep learning, Health information, Pre-trained word embeddings, Social media, Machine learning, Natural language processing, Twitter

## Abstract

This paper presents a comprehensive evaluation of data pre-processing and word embedding techniques in the context of Arabic document classification in the domain of health-related communication on social media. We evaluate 26 text pre-processings applied to Arabic tweets within the process of training a classifier to identify health-related tweets. For this task we use the (traditional) machine learning classifiers KNN, SVM, Multinomial NB and Logistic Regression. Furthermore, we report experimental results with the deep learning architectures BLSTM and CNN for the same text classification problem. Since word embeddings are more typically used as the input layer in deep networks, in the deep learning experiments we evaluate several state-of-the-art pre-trained word embeddings with the same text pre-processing applied. To achieve these goals, we use two data sets: one for both training and testing, and another for testing the generality of our models only. Our results point to the conclusion that only four out of the 26 pre-processings improve the classification accuracy significantly. For the first data set of Arabic tweets, we found that Mazajak CBOW pre-trained word embeddings as the input to a BLSTM deep network led to the most accurate classifier with F_1_ score of 89.7%. For the second data set, Mazajak Skip-Gram pre-trained word embeddings as the input to BLSTM led to the most accurate model with F_1_ score of 75.2% and accuracy of 90.7% compared to F_1_ score of 90.8% achieved by Mazajak CBOW for the same architecture but with lower accuracy of 70.89%. Our results also show that the performance of the best of the traditional classifier we trained is comparable to the deep learning methods on the first dataset, but significantly worse on the second dataset.

## Introduction

Due to the increased amount of data from user-generated content on social media, text classification has become an important area of research in the last 10 years. This has led researchers to apply text classification methods for analyzing sentiments and topics [1–3], predicting gender [4–6], and detecting false news [7, 8]. Studies on social media have indicated that, as a wide variety of people use this medium to share health information [9, 10], the information provided is not always accurate [11, 12] and this is a huge issue of concern. However, a precursor for studying the trustworthiness of health-related tweets is the development of a model to detect health-related information posts on social media.

Additional important reasons for devising a high-quality method for identifying health-information posted on social media could include building and/or studying health communication theories, evaluating health communication, and understanding public concerns on social media during an outbreak [13–15]. Studies that built models to detect (English) health information tweets were conducted by Paul et al. and Tuarob et al. [16, 17], who developed machine-learning models to detect health-related information on social media platforms.

Unfortunately, these models are highly language-dependent and, as they were not created for the Arabic language, they cannot be directly applied to this language, an important consideration given the prevalence of social media usage in Arabic countries [11]. For example, text normalization is one of the important steps in text classification. In English, this might include normalizing capital letters to lowercase letters, yet there are no lowercase and capital letters in Arabic; normalizing letters in Arabic involves normalizing different forms of alefs (**ا إ أ.** )to (ا) or removing diacritics that are not used in English. Thus, Maw et al. [18] pointed out that even if some algorithms perform well for a particular language, they might yield worse results when applied to another language.

There have been many studies of text classification regarding Arabic natural language processing on social media. Most of them are focused on sentiment analysis, and a number of literature surveys and systematic literature reviews have been conducted on this Arabic-language-classification-specific task [1–3]. More specifically, Al-Rubaiee et al. [19], Alayba et al. [20], and Alabbas et al. [21] conducted targeted sentiment-analysis studies. Al-Rubaiee et al. [19] used sentiment analysis to evaluate a bank application. They collected tweets about the bank service and labelled them as either positive or negative. They then pre-processed the tweets using various techniques and compared the performance of the Support Vector Machine (SVM) and Naïve Bayes (NB) classifiers. The best results were for SVM with an accuracy of 89.68%.

Similarly, Alayba et al. [20] collected tweets about health services in Saudi Arabia and labelled them as positive or negative. The best results were achieved using stochastic gradient descent with an accuracy of 91.87%. Moreover, Alabbas et al. [21] trained a classifier to detect natural disasters by labelling tweets, some of which contained information about a flood whereas others did not. They trained different classifiers, namely, SVM with K-Nearest Neighbors (KNN), NB, and compared their performance. The best model was SVM with an accuracy of 90.7%. Alayba and Alabbas studies are expanded on in the next section.

Other Arabic-text classification work used social media data to detect hate speech [22–24] and analyze crisis responses, such as in the event of a flood [25]. However, there is a lack of studies based on detecting Arabic-language health-related tweets. In this paper, we aim to derive a model to accurately detect Arabic language health data on Twitter and test these models on data sets to evaluate the generality thereof.

Statistics show that Twitter is very popular with Arabic speakers, and that it is widely used for sharing health-related information [9, 10]. As such, one of the goals of this paper is to enrich the literature by providing technical details for the development of a model to detect Arabic health-related tweets. Devising such a model can help researchers from many disciplines study health-related tweets in a more comprehensive manner and will provide the foundation for empirical studies that are not conducted with a focus on tweets with a specific origin only (where the origin serves as a means of determining their health-information focus by, for example, only considering tweets emanating from specific health-tweet authors/organizations). For example, while Alnemer et al. [12] extracted tweets from specific health-related Twitter accounts in order to study health-related information on social media, Albalawi et al. [11] pointed out that there are other users who also (more informally) tweet about health and that those should not be ignored in an analysis of health tweets. A model that can automatically extract health-related tweets can further the holistic study of health-related tweets without requiring that specific health-related accounts are followed. Furthermore, providing the technical details for the development of such a model will enrich the literature, not only for this specific text classification task (i.e., extracting health-information tweets), but also for other Arabic-text classification tasks.

This paper is structured as follows. First, we discuss related work in Sect. "Related work". In Sect. "Methods", we describe the general methods used in this study, focusing especially on the data sets and evaluation metrics employed. Section "First experiment" reports on the study that assesses the impact of various pre-processings on traditional machine learning techniques, when classifying health-related tweets. Subsequently, Sect. "Second experiment" describes a second study which looks at the impact of different word embeddings on deep learning algorithms for the same purpose. Finally, Sects. "Discussion" and "Conclusion" discuss and compare the results, drawing out conclusions from this work.

## Related works

There is a vast body of literature on Arabic text classification for social media. Alayba et al. [20] analyzed tweets to detect sentiment about services in Saudi Arabia. They collected tweets using trending hashtags related to health services, and then they divided their data sets into two categories: negative and positive. When processing the tweets, they removed diacritics and Kashida and normalized three additional letters: **اأإ** to **ا**., **ة** to **ه**, and **ئ** to **ى**; and they used unigram and bi-gram text extraction techniques with Term Frequency–Inverse Document Frequency, hereafter TF-IDF, for feature selection. They then compared the performance of seven algorithms and experimented with a Convolutional Neural Network (CNN). The best results were achieved with a stochastic gradient analysis and SVM, with an accuracy of 91.87. They did not use any stemming methods during pre-processing.

Alabbas et al. [21] developed a model to detect a natural disaster in tweets, specifically a high-risk flood. To achieve this, they trained a classifier on labelled tweets; some containing information about a flood and others that did not. They removed diacritics from the text based on the assumption that most text is written without diacritics. In a manner similar to that of Alayba, they used TF-IDF for feature selection. During their study, Alabbas et al. investigated the performance of different classifiers, specifically the NNET, SVM, KNN, Decision Tree (C4.5–J48), and NB algorithms. Unlike Alayba, they also compared different stemming techniques for the Arabic language: no stemming, light stemming, and prefix/suffix removal. They also normalized one letter, **اأإ** to **ا**. The authors concluded that SVM performs better than the other algorithms, and that most of the algorithms included in the study perform better without stemming.

Boudad et al. [26] compared the performance of KNN, SVM and NB in sentiment analysis for Arabic tweets. Moreover, they compared the impact of different types of stemming, specifically light stemming and root stemming; and they also compared TF-IDF to Binary Term Occurrence (BTO) for feature selection. They found that the best accuracy is achieved with light stemming, the SVM classifier, and TF-IDF for feature selection. During the normalization process, they normalized **ه** and **ى** in addition to removing hashtags. It is not obvious whether their findings contradict those of Alabbas et al. however, as the model in the earlier study was not trained without stemming.

Duwairi et al. [27] and Oussous et al. [28] studied the impact of root stemming and light stemming in addition to stop word removal on sentiment analysis. While Oussous et al. found that light stemming improves the accuracy, Duwairi et al. stated that stemming and stop word removal do not improve the accuracy of their model. Furthermore, these studies have not investigated the impact of the other pre-processing techniques discussed above. Although, Oussous et al. removed tashkeel, duplicate letters and Kashida, they did not report the impact of such steps on the results of their model.

Abdulla et al. [29] built a model to detect the sentiment of tweets. They found that light stemming decreases model accuracy, which supports the findings of Alabbas et al. In comparison to Boudad et al., however, they only normalized two letters, **ه** and **ا**. Like the studies mentioned above, they did not investigate the impact of normalizing letters on the accuracy of their model.

Alakrot et al. [24] developed a model to detect hate speech in YouTube comments, which they trained on 15,000 comments labelled as either positive or negative. They normalized the same letters as Alabbas et al. [21] along with two additional letters, because of the similar morphological sounds thereof. Their best model achieved an F_1_ score of 82%, and they reported the usefulness of stemming and normalization, which contradicts Alabbas et al. [21] and Abdulla et al. [29].

As the studies described above suggest, there is no agreement on pre-processing steps for the Arabic language as the researchers used different techniques when normalizing the text. Alabbas et al. [21] only normalized one letter, **أاإ**; Boudad et al. [26] and Abdulla et al. [29] only normalized ة **ه**; while Alayba et al. [20] and Alakrot et al. [24] normalized other letters. Furthermore, both Boudad et al. [26] and Alakrot et al. [24] reported the usefulness of stemming, while Alabbas et al. [21] and Abdulla et al. [29] found that stemming decreased the accuracy of their models. These conflicting results lead to questions as to which methods are the best for normalizing Arabic data sets, particularly for specific classification tasks.

In addition to traditional machine-learning algorithms, there has been a dramatic increase in the number of studies that apply different deep-learning methods for tackling the Arabic text classification task in the last few years. Some of these studies compared deep-learning models, such as CNN and Long Short-Term Memory (LSTM), to traditional machine-learning models. For example, Oussous et al. [30] compared four models (NB, SVM, CNN and LSTM) to detect the sentiment of tweets. They also investigated the impact of pre-processing techniques, specifically normalizing, stop-word removal, and stemming. They used traditional BTO as feature extraction for NB and SVM, and they used Word2Vec for the word-embedding layer of the CNN and LSTM models. They concluded that normalizing with light stemming improves the accuracy of their model and that the CNN and LSTM classifiers perform better than the SVM and NB ones. They only considered normalizing three letters: **ي**, **ة**, and **ا**.

It is worth noting that word embedding is a learning technique in natural language processing that represents words with vectors [31], the dimensions of which are usually set prior to the word-embedding training. A high dimension vector offers a better opportunity to represent the word semantics [22]. This technique uses geometric word encoding based on how frequently words appear together [8]; thus, words with similar meanings are represented with similar numbers. Yet, to be efficient, word embedding need to be trained on large data sets [32]. Thus, researchers often use already existing pre-trained word embedding as demonstrated by Mohaouchane et al. [33].

They [33] used the same data set that was used by Alakrot et al. [24], and they followed similar pre-processing steps to Alakrot et al.. Mohaouchane et al. [33] used AraVec pre-trained words [34] that were embedded as the input layer for a CNN, and they improved the accuracy of detecting hate speech in this data set from an F_1_ score of 82 to a score of 84.05.

In contrast to the studies by Oussous et al. [30] and Mohaouchane et al. [33], Abdullah et al. [35] developed a CNN-LSTM model to detect the emotion of tweets. Unlike Oussous et al., Abdullah et al. [35] used AraVec pre-trained words embedding for their input layer. They claimed that the normalizing and stemming steps did not improve the performance of their model.

Similar to Abdullah et al. [35], Heikal et al. [36] developed a model that uses AraVec pre-trained word embedding in the input layer. They also used different pre-processing techniques by removing diacritics, repeated characters and punctuation. They assembled a model that consisted of a CNN and LSTM architecture. The authors achieved an F_1_ score of 64%, which they claimed outperforms a state-of-the-art algorithm.

The reason the above-mentioned studies [33, 35, 36] utilized customized pre-processing techniques when using pre-trained word embeddings is unclear. According to Li et al. [37], the ideal method to achieve the most improvement when using pre-trained word embedding is to follow the same steps that were used for the corpus when creating the embeddings vectors unless they are not well-documented. The pre-processing steps to normalize the data sets when using AraVec pre-trained word embeddings are documented and were provided by the models of Soliman et al. [34].

Abuzayed and Elsayed [38] investigated the performance of classical and deep-learning models when detecting hate speech in Arabic tweets. Their results showed that the classical TF-IDF word representation performs better than word embedding with classical algorithms, but the combined CNN-LSTM deep-learning architecture performs better than the classical algorithm. This observation might help to answer the question posed by Guellil et al. [39]: “Are deep-learning approaches really more efficient than traditional approaches, such as SVM, NB, etc., for Arabic natural processing?” (p. 9). This is a core research agenda for this work, but in the context of classifying/identifying health tweets in particular.

While Mohaouchane et al. [33], Abdullah et al. [35], and Heikal et al. [36] used AraVec pre-trained words embedding, there are additional pre-trained Arabic word embedding models that have been investigated. Alwehaibi and Roy [40] asserted that pre-trained models require millions of words to be effectively trained; consequently, they investigated the usefulness of the AraVec, fastText, and the ‘Altowayan and Tao’ [41] pre-trained word-embedding techniques for text classification. To compare these classification approaches, they developed a CNN-LSTM deep neural network model to predict the sentiment of tweets, and they found that the Altowayan and Tao [41] pre-trained word-embedding method outperforms AraVec and fastText as the authors’ best model achieved 93.5% accuracy when classifying texts into positive, negative and neutral sentiment.

Utilizing a collection of 55 million tweets, Fouad et al. [42] developed their own pre-trained word-embedding model by combining three popular techniques—Word2Vec Skip-Gram; Word2Vec Continuous Bag-of-Words (CBOW); and Global Vectors (GloVe). Using the CNN architecture, they compared the performance of their pre-trained word embeddings (ArWordVec) to that of AraVec pre-trained word-embedding methods and found their pre-trained model outperformed the AraVec model.

Based on the literature identified above, Table 1 presents the pre-trained word-embedding models that have been applied to the classification of Arabic texts.

**Table 1:** Pre-trained word embedding models

Pre-trained word embedding	Number of documents	Sources	Techniques	Availability	Pre-processing
fastText [45]	400 millions tokens from Wikipedia”, i.e. 400 million Wikipedia articles + “24 terabytes of raw text data” from Common Crawl	Common Crawl and Wikipedia	CBOW with sub-wording techniques applied to the methods	Open	Only tokenization
AraVec [34]	66.9 million tweets and 320,636 documents from Wikipedia	Twitter and Wikipedia	CBOW and Skip-Gram with different n-gram and unigram features	Open	Remove non-Arabic letters. replace ة with ه. Normalize alef. remove duplicates, Normalize mentions, URLs emojis
Mazajak [46]	250 million tweets	Twitter	CBOW and Skip-Gram with different n-gram	Open	Removal URLs, Tashkeel, emojis and punctuation
ArWordVec [43]	55 million tweets	Twitter	CBOW and Skip-Gram	Open	Normalize mentions, URLs. Remove tashkeel, punctuation, Normalize bare alef Replace ى" with "ي", Replace ؤ" with “ء", Replace ئ" with ء", Replace " ة with ه "

It is worth noting that the majority of the studies reviewed above, which used Arabic social media for text classification tasks, used SVM followed by NB. There is also a recent trend of using deep-learning methods for Arabic text classification, where CNN and LSTM architectures were primarily used as deep learning methods. This observation is consistent with the findings of Oueslati et al. [43], who conducted a review on the techniques used for sentiment analysis of Arabic-language tweets.

While several recent studies reported the effectiveness of using pre-trained words as the embedding layer for deep-learning models, there have been only few comparative studies of word-embedding techniques in the context of Arabic text mining. For example, four different studies [33, 35, 36, 38] used AraVec, only, one study used fastText [41], and no studies were found that used ArWordVec.

As for traditional methods, the majority of Arabic works have emphasized some pre-processing techniques, such as stemming, but none of the studies discussed determined the impact of normalizing Arabic letters or removing diacritics. Some claimed these techniques negatively affect the classifier performance [44, 45] but did not elaborate on or provide evidence for their assertions. Furthermore, there have been no studies to date on the detection of Arabic health-related tweets on Twitter.

Thus, this paper aimed to investigate the impact of different pre-processing techniques on model accuracy. An additional aim was to employ deep-learning methods to compare the performances of pre-trained word-embedding techniques. This will be carried out through a text-classification task focused on detecting Arabic-language health-related tweets. Using these studies as pre-requisites, this study aimed to compare the best classifiers developed using deep learning methods to best classifiers developed using traditional machine learning (ML) methods to identify the overall best-of-breed classification approach available for health tweet identification.

## Methods

We derived two different approaches to achieve the aims of the study.

For the first objective, which concerned testing the impact of pre-processing techniques on the accuracy of the predictive models, we tested 14 variants of normalizing Arabic letters in addition to 12 pre-processing techniques (explained in Sect. 4.1) on four different algorithms. These algorithms are among the most widely used algorithms for text classification [46].

In the second experiment, which was performed to answer the second objective of this study (comparing the performance of the CBOW/Skip-Gram variants of the four pre-trained word embeddings presented in Table 1 using a deep learning approach) we specifically used CNN and the BLSTM (Bidirectional LSTM) architecture to compare these pre-trained word embedding models. BLSTM and CNN are among the most used deep learning architecture that have been applied to text classification problem [47, 48].

**Fig. 1:**
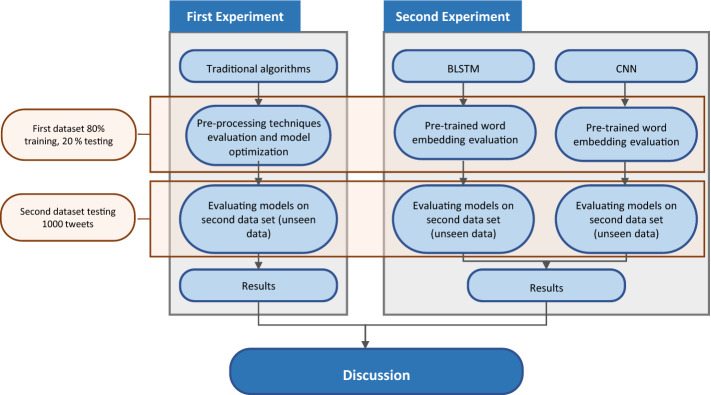
Study overview

Lastly, we compared the accuracy of classifier models developed using traditional ML methods to classifier models developed using deep learning methods. Figure 1 presents an overview figure for this study.

### Data sets and model evaluation metrics

Generalization is the ability of a trained model to accurately categorize new data for which it has not been previously seen/exposed [49]. Chung et al. [50] ⁠state that even though most machine-learning-development data is divided into testing and training examples it is questionable whether a machine-learning model would hold in a more general sense as both the training and the test data sets are usually derived from the same environment. Thus, in addition to a first data set, on which each model was trained and tested, we further tested each model on another data set on which the models had not been trained. This data set included words related to COVID-19 and was extracted between March 2020 and April 2020. It differs from the first data set in two ways. Firstly, it was created at a different time-point and secondly, it was extracted during a pandemic, which allowed the model to be tested more for generally.

Next, we describe the process of creating the health lexicon used in extracting these data sets, and then provide more details about each data set.

#### Health lexicon

The health lexicon, used for extracting health-related tweets, combines keywords from three different sources in order to minimize bias [51]. These sources include are:

- an annotator—a graduate linguist and native Arabic speaker who reviewed health-related accounts and hashtags to identify 110 health-related words.
- field experts—three medical doctors who are active on Twitter; they suggested 100 health-specific words that typically occur in health-related tweets.
- an existing health dictionary—we took 232 words from the Arabic health dictionary proposed by Collier et al. [52]. These 232 words are the only words out of all 968 words in the dictionary, that occur in the tweets we have collected.

We then combined all the words. However, we found that there were still some words not *specific* to health in the lexicon, resulting in a high number of false positive tweets. Thus, similar to Hicks et al. [53], Prus et al. [54] and Zhang and Ahmed [55], we removed these words. Our final lexicon consists of 263 Arabic health-related terms created from the sources above. It is available at http://tiny.cc/ArabicHealthLexicon.

#### The first data set

Using the health lexicon described above, 297,928 tweets were collected (by employing the Twitter Premium API). These are tweets posted between the 15th July and the 31st August 2019. 5000 tweets were randomly sampled from the data set. These tweets were independently classified by two annotators as either “health-related” or “not health-related.” By following Shoukry and Rafael’s [56] procedure, a third annotator was brought in whenever there was a disagreement between the two annotators.

Cohen’s kappa statistic for interrater reliability [57] demonstrated excellent agreement between the two annotators independent coding (k = 0.84). As a result, 1,415 of the 5000 tweets (28.3%) were labelled as health related. Both models were trained on 80% of this data set and were tested on the remaining 20%. This data set is available at http://tiny.cc/AlbalawiDS1.

Data set imbalance typically needs management [58]. At the algorithms level for traditional machine learning, we tried different models, as explained in Sect. 4.3. For example, one of the algorithms we used, SVM, is known to be less impacted by imbalanced data [59].

Another solution to handle an imbalanced data set is to rework the data sets by re-sampling. However, reworking the data sets in this fashion would increase complexity, and is not guaranteed to increase the model’s performance [60, 61]. In addition, the data set that we used is only slightly imbalanced, with a ratio of 1.2:3. Sun et al. [59] states that an imbalanced data set is one having “many more instances of certain classes than others” with Somasundaram et al. [62] going further to suggest that “a dataset is considered to be imbalanced if one of its classes plays a huge dominance over the rest of the classes.” The imbalance in our data set is not of that scale and Brownlee [63] states that slightly imbalanced data sets should not be a concern: that typically such a classification problem should be treated as classification problem with a balanced dataset.

#### The second data set

The second data set used in this study consists of tweets posted between the 20th February and the 31st March 2020. First, we extracted 4,548,839 Arabic tweets using COVID-19-related keywords and then applied our health lexicon to reduce the number of tweets. Finally, we sampled and manually labelled 1,000 tweets from this data set, which is the same number of tweets used for test from the first data set. In this sample, 188 tweets are labelled as health related. We refer to this data set as *unseen data* in this paper as it was not used in any way for training or evaluation of classifiers and it is collected from a different time period compared to the first data set. This data set is available at http://tiny.cc/AlbalawiDS2.

Please note we only share tweet IDs and the labels as the Twitter policy prevents the content of the tweets to be redistributed, apart from tweets IDs, that can be used to obtain the text of the tweets with the Twitter API [64].

### Evaluation metrics

To evaluate the traditional algorithms, we used the F_1_ score. The F_1_ score is a recommended metric for imbalanced data sets, while accuracy is the recommended metric for a balanced data set [63]. To evaluate the final model in both experiments and make the comparison between them, we used four metrics—recall, precision, F_1_ score, and accuracy. These are the most-used metrics to evaluate machine-learning model performance [3, 65]. Yet, as per the first experiment and the recommendation of Brownlee [63], we used F_1_ score as the decisive metric to select the best-of-breed model.

## First experiment

The first experiment concerned traditional ML algorithms. It evaluated the importance of different pre-processing techniques and their impact on classification.

### Common pre-processing techniques

By reviewing the literature, we were able to identify more than 26 pre-processings for potential analysis: 14 variants of normalizing Arabic letters in addition to 12 techniques have been applied in the pre-processing steps on Arabic-language social media data:

#### Tokenization

In the tokenization process, text is divided into units, and typically here, those units are words. They are usually delimited by spaces or punctuation, and the results are referred to as tokens [66].

#### Noise removal

Noise removal aims to eliminate unwanted characters from the text. In the literature, we found the following techniques used:

##### Removal of non-Arabic letters

Several of the aforementioned studies [21, 35, 67–69] removed non-Arabic data from the text examples.

##### Removing numbers

Numbers do not always contribute additional information about the text. We found three studies [70–72] that emphasized number removal from the sampled texts.

##### Removing usernames, external links, and hashtags

Usernames, external links, and hashtags are found in many tweets. Three of the cited studies [73–75] removed these from the text.

#### Normalization

Normalization is a process that converts a list of words to a more uniform sequence [22]. In the literature, we found 5 techniques used for this:

##### Removal of punctuation

Punctuations marks typically do not add extra meaning to the text, although punctuation sometimes has a useful meaning, especially when analysing sentiment [76]. Eight of the previous studies [35, 67, 68, 77–81] removed the punctuation from the examined text.

##### Removal of tashkeel (diacritics)

Diacritics are often used to represent short vowels, gemination or nunation [82]. In Arabic there are 8 basic diacritics and if combined they can form a total of 13 different diacritics [83]. Four of the previously described studies [40, 75, 84, 85] removed diacritics.

##### Removing repeated characters

Because some users use repeated characters when they want to emphasize something, researchers refer to this as the speech effect. Several of the cited studies [67, 69, 72, 86, 87] removed these characters.

##### Removal of duplicate letters

The rationale for these removals is similar to that for removing repeated characters. However, some argue that many Arabic words originally contains repeated letters, so they only deleted characters if they occur more than twice. An example of this is the work of Alqarafi et al. [80], who deleted duplicate letters if they occurred more than twice.

##### Removing Kashida

Kashida, also known as *tatweel*, is a decorative element in Arabic writing used to justify or stretch the text with a phonetic value [88]. We found two studies [36, 45] that removed Kashida.

#### Arabic-specific normalization

Arabic is considered a Semitic language, with script written from right to left. The Arabic language has 28 letters. However, as some Arabic letters are phonetically similar, users on social media frequently misspell words by using the wrong but phonetically similar letters [24]. In addition to some phonetically similar letters, some letters can be written in more than one form. This might be more apparent in the case of the alef variances “أإآ”, which are often written as a bare alef “ا”; possibly due to their similarity in appearance [89, 90]. For example, the word “أنت”, which means 'you' in modern standard Arabic, is commonly written as “انت”, i.e. without the hamza “ء”, and some people might even misspell it and write it as “إنت”, with the hamza under the alef [91]. Thus, different forms of alef are unified as a bare alef.

Hence, Arabic-specific normalization indicates that the normalization is specific to the Arabic language as it directly deals with Arabic letters; therefore, it is not possible to apply these Arabic-specific normalization techniques to other languages. In the literature, some researchers have normalized two letters, while others have normalized five or six letters. Furthermore, the same letters are sometimes normalized in different ways. For example, “ي” and ئ” have been replaced with “**ى**” [92], and “ىء” and “ئ” have been replaced with “ي” [78]. Table 2 summarizes the most-used techniques for normalizing Arabic letters that were presented in the literature.

**Table 2:** Normalization techniques used by different researchers

Replace	With	Relevant studies
**أ**, **إ**, and **آ**	Bare-alif **ا**	[21, 24, 26, 71, 74, 92–97]
**ى**	**ي**	[23, 26, 78, 84, 93–98]
**ي** and **ئ**	**ى**	[92]
**ىء** and **ئ**	**ي**	[78]
**ؤ** and **ئ**	**ء**	[77, 94, 96, 99, 100]
**ئ**	**ى**	[85]
**ة**	**ه**	[20, 74, 85, 94–97, 99–101]
**چ**	**ج**	[100]
**ڤ**	**ف**	[100]
**ءى** and ءي	**ئ**	[71]
**ص**	**س**	[24]
**ض**	**ظ**	[24]
**ؤ**	**و**	[71, 78, 99]
**كـ**	**ك**	[38, 77]

Please note that this study does not aim to be conclusive regarding all possible pre-processing techniques. It focuses on pre-processing techniques commonly identified in our literature review, and only those techniques. While this is not entirely systematic, it does provide high coverage of work-to-date and it improves on current research practice where the basis for selecting pre-processing techniques is not presented explicitly [27, 28, 76]. However, future work should consider this issue carefully, to incorporate additional, relevant pre-processing techniques not yet considered, or only tangentially considered, by the community.

#### Removing stop words

Many studies removed stop words. There are several methods of removal for Arabic stop words. Examples of studies that removed stop words are [19, 35, 93].

#### Stemming

Stemming is the process used to get the stem from the word. To achieve this, three different techniques are used in the literature:

##### Light stemming

Light stemming is the process of removing the prefixes, infixes and clitics from words. For light stemming, we used the Tashaphyne Python library [102]. This method was used in three of the cited studies [19, 100, 103].

##### Root stemming

Root stemming, which is also called also heavy stemming, aims to transform a word to its root [83]. It is usually faster to perform than lemmatization (see Sect. 4.1.6.3). In Arabic, most word roots consist of three letters [104]. Thus, the results of root-stemmed words will be mostly words made from three letters. For the root stemming, we used the Tashaphyne Python library [102].

##### Lemmatization

Lemmatization has a similar aim as root stemming in that the aim is to return a word to its origin; however, unlike root stemming, lemmatization uses a lexicon or dictionary to map a word to its root. Thus, in the present study, to get the roots of Arabic words, we mapped a word to its roots using the dictionary Qalsadi [105].

### Feature extraction

The feature-extraction process transforms text into vectors [106]. Bags of words (BOW) and TF-IDF are the two most-used methods for extracting features from the text. In BOW, words frequencies are counted, and word position is ignored. TF-IDF is considered to be a statistical approach that is more sensitive for less-general words as TF measures term frequencies in the text, and IDF is a proxy for the importance of a term [107].

### Classification algorithms used

#### Multinomial NB

NB is a probabilistic model, and in its basic version it is one the most-used algorithms in text classification [108], including sentiment analysis [80, 109] and spam filtering [110]. In this work, we used the variation of NB knows as Multinominal NB (MNB) [111].

#### SVM

SVM, which is grounded in statistical learning theory, is one of the most popular ML classification methods.

SVC and NuSVC are implementations of support vector machine classifiers. They are quite similar and are both based on LIBSVM (Library for SVMs), which was devised by Chang and Lin [112]. LinearSVC is based on the work of Fan et al. [113] and is more flexible than SVC because it provides more options for penalties and choices of loss functions [114]. In this study, we used LinearSVC.

#### Logistic regression

Logistic regression is a linear classifier that uses a hyperplane to separate two classes. This algorithm was used in the present study to differentiate between health-related and non-health-related tweets in accordance with the work of Dressel and Farid [115].

#### KNN

KNN, is fundamentally different from other algorithms discussed in this paper because this algorithm memorizes the training data set rather than learning discriminative functions, and it is thus classified as a memory-based approach [116].

### Experiment setup and results

**Fig. 2:**
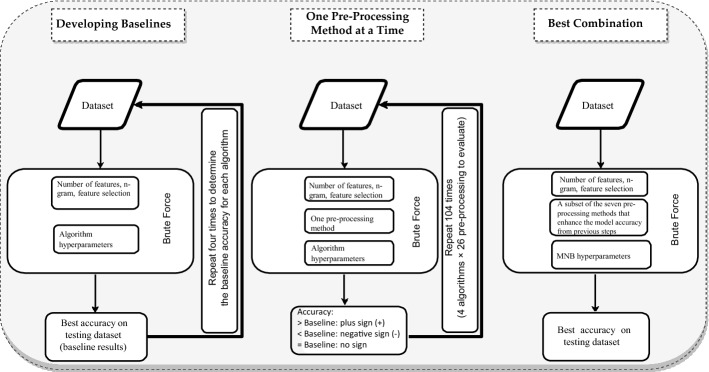
Overview flowchart for the first experiment. (In the best combination, we only tried MNB as it was the best algorithm from previous steps.)

The setup of the first experiment consists of three phases. The baselines for each algorithm were first developed. Each pre-processing technique was then individually tested on each of the four algorithms, and the results were compared against the baseline for each algorithm. It would be computationally expensive to apply all combinations of pre-processings for each algorithm. Thus, the approach followed in this study is to evaluate each pre-processing technique with the four selected algorithms. We then apply the combination of pre-processing techniques that best enhance the model performance in the second phase, using brute force to combine the pre-processing techniques and find the best combination. Lastly, we choose the best model with the best combination to evaluate on the second data set. Figure 2 illustrates these three phases and the flow of the first experiment.

#### Developing baselines

We trained four baselines models without applying any of 26 pre-processings. Moreover, we use Python Grid Search and Pipeline to tune hyperparameters as well as to apply other pre-processing techniques that this paper does not assess, which are outlined in Table 3. Each algorithm has a number of hyperparameters that must be tuned, so they are also “brute-forced”. The hyperparameters for each algorithm are outlined in Appendix 1: Tables 9, 10, 11, and 12

**Table 3:** Techniques used as brute-force algorithms in each attempt for all algorithms

Techniques used	Parameters or range used
Number of features	Ranges from 7,000 to 18,000
N-gram	(1, 1), (1, 2), (1, 3), (1, 4)
Feature selection	Count vectorizer and TF-IDF

Table 4 outlines the accuracy results achieved for the testing data set without applying any pre-processing methods that this paper aimed to investigate. Hence, these models were used as baselines to compare to the impact of pre-processing. It is important to note that these were not used as a standard for further development; instead, we used the best achieved accuracy as the baseline to judge whether other pre-processing methods improve the results or not.

**Table 4:** Baseline results for four algorithms used in this study

Algorithm	N-gram	Feature selection	F_1_ score
LinearSVC	1	Term frequency	84 .0
Logistic regression	1, 2	TF-IDF	84.0
Multinomial NB	1, 2	TF-IDF	86.0
KNN	1, 2	TF-IDF	77.6

Finally, we utilized four cross-validations during model development. We used Python and the scikit-learn Version 0.22 library to conduct these experiments [117].

#### Using one pre-processing method at a time

The accuracy of each algorithm without applying pre-processing techniques is used as a baseline to compare with the performance of each of the pre-processing methods discussed in Sect. 3.1. We employed methods that are similar to those used by Symeonidis et al. [76], who compared the impact of these pre-processing techniques on classifiers trained for the sentimental analysis of English language. We applied one technique at a time, applied to each of the four classification algorithms. The model that achieved the most accurate results was then selected for further refinement, with all the pre-processing combinations that were shown to enhance the accuracy of the model presented in Table 5.

**Table 5:** Accuracy (in percentages) of each of the pre-processing techniques used for the extracted tweets

	Techniques used	MNB	Logistic regression	LinearSVC	KNN
	Baseline models	86.0	84.0	84.0	77.6
1	Remove non-Arabic letters	85.4 −	83.6 −	82.8 −	76.7 −
2	Remove numbers	85.5 −	82.9 −	84.3	77.4 −
3	Remove usernames, external links, and hashtags	85.2 −	83.2 −	83.4 −	78.1 +
4	Remove punctuation	86.0	84.0	84.0	77.6
5	Remove diacritics	86.0	83.6	83.8 −	76.6 −
6	Remove repeated characters	86.4 +	84.3 +	84.9 +	79.2 +
7	Remove duplicate letters	86.0	83.2 −	84.1 +	78.7 +
8	Remove Kashida	86.3 +	83.8 −	84.6 +	78.0 +
9	Replace **أ**,**إ**, and **آ** with **ا**	85.8 −	83.6 −	84.1 +	77.4 −
10	Replace **ى** with **ي**	86.7 +	84.0	84.6 +	77.9 +
11	Replace **ي** and **ئ** with **ى**	86.8 +	84.0	84.2 +	78.0 +
12	Replace **ىء** and **ئ** with **ي**	86.0	83.0 −	84.3 +	77.8 +
13	Replace **ؤ** and **ئ** with **ء**	85.8 −	83.8 −	83.9	77.7 +
14	Replace **ئ** with **ى**	86.0	84.0	84.3 +	77.6
15	Replace **ة** with **ه**	86.7 +	83.8 −	84.8 +	77.1 −
16	Replace **چ** with **ج**	86.0	84.0	84.0	77.6
17	Replace **ڤ** with **ف**	86.0	82.8 +	84.0	77.6
18	Replace **ءى** and **ءي** with **ئ**	86.0	84.0	84.0	77.6
19	Replace **ص** with **س**	85.7 −	83.7 −	83.6 −	78.0 +
20	Replace **ض** with **ظ**	86.0	83.6 −	84.0	77.9 +
21	Replace **ؤ** with **و**	85.8 −	82.8 −	84.2 +	77.6
22	Replace **كـ** with **ك**	86.0	84.0	84.2 +	77.6
23	Remove stop words	85.2 −	84.4 +	83.4 −	76.6 −
24	Light Stemming	86.6 +	85.3 +	86.2 +	79.1 +
25	Root stemming	84.4 −	85.2 +	85.1 +	77.8 +
26	Lemmatization	86.7 +	86.2 +	86.5 +	80.1 +

Plus sign ( +) indicate the technique improved the F_1_−score of the baseline model; negative sign(−) indicate the technique decreased the F_1_-score; and cells without sign indicate the technique had no impact on the F_1_-score of the algorithm

Take, for example, the seven techniques that enhance the MNB classifier. All the possible combinations of those seven were calculated by the following equation ${2}^{n},$ where n is the number of pre-processing techniques. Therefore, we tried 128 variations as ${2}^{7}=128$. The results of these experiment are found in Appendix 2.

As mentioned above, the results show that 7 pre-processing and normalization techniques improved the MNB and logistic regression performance in terms of F_1_ score, 15 techniques improved LinearSVC and 13 improved KNN. It is worth noting that Light Stemming, Lemmatization and Remove repeated characters improved the F_1_ score in all the models we tried, whereas Remove non-Arabic letters reduced the F_1_ score in all the models, as shown in Table 5.

#### Best combination

In the third phase, we used a brute-force algorithm to determine the best combinations of the favourable pre-processing techniques discussed above. This phase focused on MNB, as it achieved the best performance for all but one variant in the previous phases.

It is worth mentioning that, for the MNB model, not all the pre-processing techniques listed in Table 5 as favourable were shown to be the most effective in combination. For example, out of the seven pre-processing techniques that improved the MNB classifier, only four contributed to the best combination. In other words, after experimenting with all the combinations, we found that MNB achieved the best F_1_ score with a combination of remove duplicate, remove Kashida, replacing ة with **ه** and replacing ى with ي. This combination improved the F_1_ score from 86.0% to 87.9% on the first data set. In terms of generalization, when we applied the best model on the second data set, the accuracy of the algorithm sharply decreased to 60.54%, which might be due to the fact there were words included that the algorithms had not seen before. These results are shown in Table 6.

**Table 6:** Results for MNB classifier with the best combinations

Model	Baseline Classifier				Optimized classifier			
Metrics	Precision	Recall	F_1_ Score	Accuracy	Precision	Recall	F_1_ Score	Accuracy
First Dataset	87.5	84.6	86.0	91.6	89.1	86.6	87.9	92.7
Second Dataset	61.5	55.0	58.1	85.1	66.5	55.6	60.5	86.4

## Second experiment

In the second experiment, we aimed to investigate four pre-trained word embedding models for Arabic found in the literature using deep learning methods. These pre-trained models were summarized in Table 1 above. We also aimed to compare the best classifier model produced in this experiment to the best classifier model produced using tradition ML methods, as a result of the first experiment.

In the second experiment, we trained a classifier using a deep-learning approach. As this work sought to generalize a model for new data, we use pre-trained words as the input layer for the model. According to the literature and, as described in an earlier section, there are four pre-trained word embedding models, all of which are found in Table 1.

Using trained word-embedding models provides an opportunity for the classifier to correctly classify words that were not seen in the training data set [118], which solves the problem in traditional text classification that occurs when the classifier fails upon encountering an unseen word [119]. For pre-processing text in the second experiment, we employed the same steps provided by the authors of pre-trained word embeddings models. According to Li et al. [37], the ideal method to achieve the most improvement when using pre-trained word embedding is to follow the same steps that were used for the corpus when creating the embeddings vectors.

### Models

We experimented with BLSTM and CNN architectures for the classification task in order to compare the different pre-trained word-embedding techniques.

#### BLSTM

**Fig. 3:**
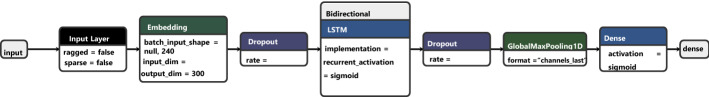
BLSTM architecture

Assuming that the input to a neural network is a sequence of data, LSTM is a type of recurrent neural network that is designed to learn and take advantage of dependencies between parts of the input sequence. Text is a sequence of words, and the LSTM architecture has been found to give good results in text classification tasks, specifically in its BLSTM variation, which learns dependencies on both past and future elements in the input sequence [120]. In this work, we experimented with an BLSTM architecture similar to the one proposed by Soufan [121]. The BLSTM model begins with an input and embedding layers to which a dropout layer is added, and this is followed by the BLSTM layer with an added dropout layer. To reduce the dimension of output from this model, a global max-pooling layer is used, as shown in Fig. 3

#### CNN

**Fig. 4:**
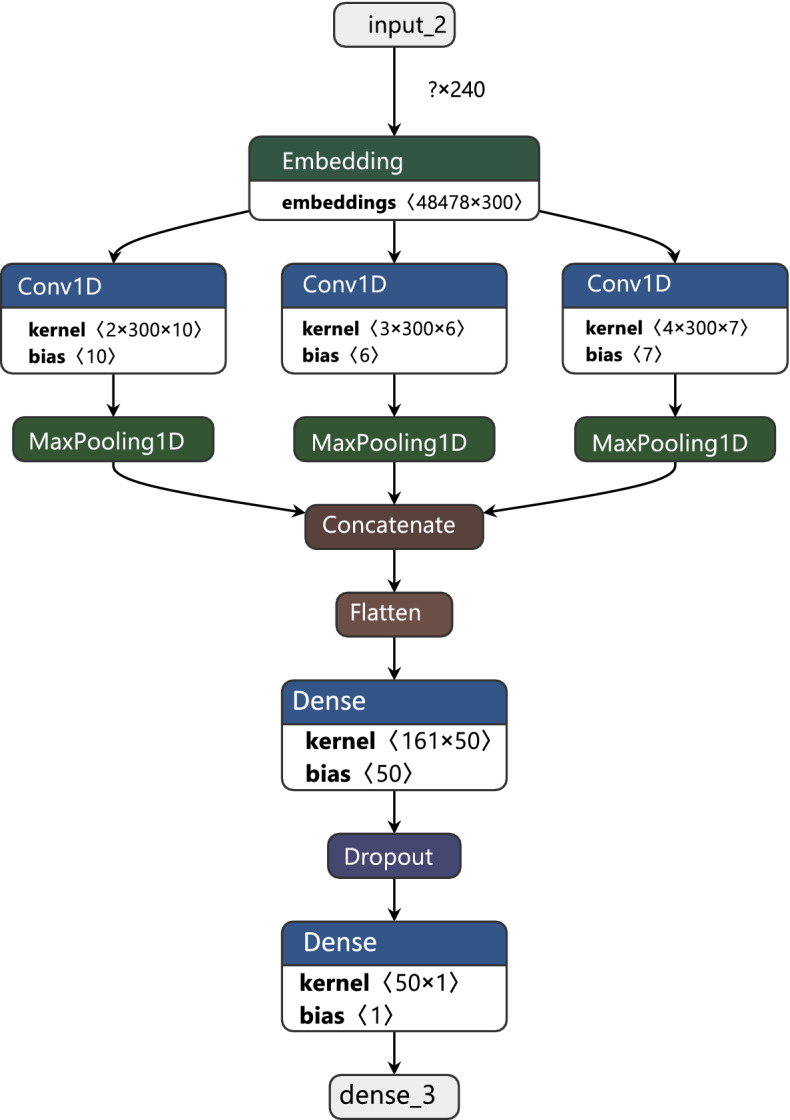
CNN architecture

While CNN was originally proposed for image analysis, this deep learning architecture was recently proven to perform effectively on many text classification problems; in fact, it sometimes performs better than other approaches, including BLSTM [33, 122]. In this work, we proposed an architecture that is similar to that of Mohaouchane et al. [33], Heikal et al. [36] and Fouad et al. [42]. The proposed model begins with input and embedding layers followed by three CNN layers, each of which contains input and embeddings from the previous layer. Max-pooling layers are used after each of the CNN layers to reduce the output dimensions, and all output from these layers is concatenated and flattened before including a fully connected layer. Figure 4 illustrates the architecture of the CNN model used in this study.

#### Hyperparameter tuning

There are different hyperparameters that must be tuned to optimize the performance of the model. Several methods are suggested in the literature, including random search, grid search, and the Bayes method [123, 124]. According to Hutter et al. [123] and Feurer and Hutter [124], the Bayes method outperforms other tuning methods. We therefore used the implantation of this algorithm in the Keras Tuner Python library [125]. We limited each experiment to 200 rounds, and the model was terminated if the rounds did not achieve optimal results, with only the best results then used. Appendix 3: Table 13 outlines the best hyperparameters for the BLSTM model, and in Appendix 3: Table 14 outlines the best hyperparameters for the CNN model.

## Results

Here we present the results of seven pretrained word embeddings that were used as input layers for two architectures BLSTM and CNN, as shown in Table 1.

### First model: BLSTM

It can be observed from Table 7 that, for the first data set, most of the pre-trained word-embedding models caused the BLSTM to perform in a similar manner. The highest accuracy and F_1_ score achieved by BLSTM, with Mazajak CBOW, were 93.8% and 89.7%, respectively. Mazajak CBOW also achieved the second-best recall and precision at 90.9% and 88.52% respectively. The highest precision was achieved by ArWordVec CBOW at 91.87%, while the highest recall was achieved by AraVec Skip-Gram at 88.85%. It is also noted that the performance of AraVec CBOW was the worst in terms of precision, F_1_ score and accuracy with results at 87.09%, 86.66% and 91.9%, respectively.

**Table 7:** Results of BLSTM using different pre-trained word embeddings on the first and second data sets

	First data set				Second data set			
	Precision	Recall	F_1_ score	Accuracy	Precision	Recall	F_1_ score	Accuracy
AraVec Skip-Gram	89.14	**88.85**	89	93.3	82.19	63.49	71.64	90.5
AraVec CBOW	87.09	86.23	86.66	91.9	79.35	65.08	71.51	90.27
Mazajak Skip-Gram	90.27	88.2	89.22	93.5	75.81	**74.6**	**75.2**	90.7
Mazajak CBOW	90.9	88.52	**89.7**	**93.8**	**88.19**	59.26	70.89	**90.8**
fastText	89	87.54	88.26	92.9	83.2	57.67	68.13	89.8
ArWordVec Skip-Gram	89.6	87.54	88.56	93.1	71.76	64.55	67.97	88.5
ArWordVec CBOW	**91.87**	85.25	88.44	93.2	76.92	68.78	72.63	90.2

Bold numbers indicate the best value while underlined numbers represent the second-best value

Similarly, on the second data set, it is shown in Table 8 that Mazajak CBOW had the best precision and accuracy at 88.16% and 90.8%, respectively. Mazajak Skip-Gram performed similarly to Mazajak CBOW on the second data set and achieved the best recall at 74.6% and the best F_1_ score at 75.2%. Mazajak Skip-Gram achieved the second-best accuracy at 90.7% as compared to 90.8% achieved by Mazajak CBOW, a difference of only 0.1%. Overall, it is noted from Table 7 that the best pretrained word embedding model using BLSTM architectures for both data sets is Mazajak Skip-Gram as it is had the second best F_1_ score on the first data set and the best F_1_ score on the second data set. As explained in Sect. 2.2, the F_1_ score was used as a judgment metric as F_1_ is more optimal for imbalanced data set [63].

**Table 8:** Results of CNN model using different pre-trained word embeddings in the first and second data sets. Bold numbers indicate the best value while underlined numbers represent the second-best value

	First data set				Second data set			
	Precision	Recall	F_1_ score	Accuracy	Precision	Recall	F_1_ score	Accuracy
AraVec Skip-Gram	88.16	**87.87**	**88.01**	**92.7**	76.84	**71.96**	**74.32**	**90.6**
AraVec CBOW	87.46	84.59	86	91.6	78.31	63.49	70.18	89.8
Mazajak Skip-Gram	89.04	85.25	87.1	92.3	78.26	66.67	72	90.2
Mazajak CBOW	89.44	83.28	86.25	91.9	81.05	65.61	72.51	90.6
fastText	87.46	84.59	86	91.6	**84.12**	56.08	67.3	89.7
ArWordVec Skip-Gram	85.76	82.95	84.33	90.6	70.56	67.2	68.83	88.5
ArWordVec CBOW	**89.67**	79.67	84.38	91	78.57	64.02	70.55	89.9

Bold numbers indicate the best value while underlined numbers represent the second-best value

### *Second model*: CNN

In contrast, as shown in Table 8, for the first data set, AraVec Skip-Gram had the best CNN performance with an accuracy of 92.7%, and F_1_ score of 88.01% and recall at 88.87%, as shown in Table 8. The best precision was achieved by ArWordVec CBOW at 89.67%. The second-best model performance was Mazajak Skip-Gram for recall, F_1_ score and accuracy at 85.25%, 87.1% and 92.3%, respectively.

For the second data set, the best performance model was again AraVec Skip-Gram, with 71.96% for recall, 74.32% for F_1_ score and 90.6% for the accuracy, while fastText achieved the best precision at 84.12% but had the worst recall and F_1_ score at 56.08% and 67.3%, respectively.

When comparing pre-trained embedding models performance using the two architectures, the AraVec performance with either Skip-Gram or CBOW did not change significantly between the two architectures, while the other pre-trained word embeddings Mazajak and ArWordVec both decreased. This caused AraVec Skip-Gram to perform better using CNN architecture. Thus, to choose the overall best model for the CNN architecture, it was found that CNN with AraVec Skip-Gram performed the best in terms of the F_1_ score on the first and the second data sets, as shown in Table 8. In addition, most of pre-trained word-embedding models performed better with BLSTM architecture; therefore, BLSTM generally appears to perform better when detecting Arabic-language health-related tweets in this study. This is particularly true with its best embedding (Mazajak Skip-Gram) for both first and second data sets.

## Discussion

For the first experiment, which was concerned with pre-processing techniques, the best algorithm performance was achieved with 4 pre-processings out of a possible 26. Some of the popular techniques presented in Table 5 used by other researchers, such as normalizing *alef* and *different types of stemming* and removing numbers were not pre-processing methods that improved the accuracy of our final model in the first experiment.

In the literature, there was a focus on studying the impact of stemming on algorithm performance [24, 26, 28, 84]. Most studies found stemming increased the accuracy of the baseline model [24, 26, 84], and this study is in agreement with theses previous studies. Having said that, the best combination of the pre-processing techniques for our final model outperformed any combination of pre-processing that included any type of stemming, as shown in Appendix 2. It also important to note that, out the four pre-processing techniques that were used in the final model, only one can be considered as not being an Arabic specific pre-processing technique, which is the removal of the repeated character, Normalizing the letters ي andه are Arabic specific. Likewise, the fourth pre-processing technique removed Kashida, which is widely used by Arabic writers. This might suggest that in text classification for the Arabic language, Arabic specific normalization techniques might play a bigger role in improving the model performance compared to the other general pre-processing techniques. This possibility also highlights the importance of this study and the need for more studies to systemically assess the impact of normalizing Arabic specific techniques on the model performance of more data sets.

Nevertheless, we found that rarely used pre-processing techniques performed well in improving the classifier model. For example, lemmatization was only used in one study [46] in the literature reviewed in this paper. Yet, as it can be seen in Table 5, lemmatization performed well with all four classifier models. Notwithstanding, it was not one of the four techniques that improved the accuracy of the final best MNB model in the first experiment. It is also worth noting that whereas the MNB classifier achieved an 87.7 F_1_ score on the first data set, its performance decreased on the second data set.

In the second experiment, we noted two observations. Firstly, there was no big difference between Mazajak Skip-Gram and Mazajak CBOW in their performance on the first data set using BLSTM. This also applied for Mazajak Skip-Gram and CBOW with BLSTM on the second data set. Furthermore, this also applied for Mazajak and ArWordVec using the CNN architecture. In contrast, there was a noted difference when we compared the performance of AraVec CBOW to Skip-Gram: AraVec Skip-Gram performed better than AraVec CBOW in both architectures. The second observation is the AraVec performance slightly decreased between the two architectures, whereas the Mazajak, ArWordVec and fasText had a more notable decrease. This caused AraVec to perform better using CNN architecture than other pretrained word embedding models on the first data set. Furthermore, on the second data set using the CNN architecture, AraVec Skip-Gram performance had a negligible increase compared to BLSTM architecture.

When comparing deep-learning methods to traditional algorithms, the results for the first data set indicated that the BLSTM architecture with all pretrained model embeddings performed better than the MNB classifier except for AraVec CBOW, where the MNB classifier performed better. When models using the CNN architecture, were compared to the MNB classifier, it is found that the MNB classifier performed better than most CNN classifiers, except for the CNN classifier that used AraVec Skip-Gram as an input layer, as is reported in Tables 6 and 8. The CNN classifier that used AraVec as an input layer performed identically in terms of accuracy at 92.7% and only marginally different for F_1_ score at 88.01% compared to 87.9%, where AraVec Skip-Gram performed better than the MNB classifier.

In the second data set, however, the CNN and BLSTM models both performed better with all the pre-trained word-embedding models than did the MNB classifier. The results suggest that the MNB classifier for the first data set is comparative to some deep learning methods, but all the deep learning methods outperformed the MNB classifier on the second data set, this data set representing more generalized, unseen data. This might contribute to answering the question in the literature that Guellil et al. posed [39]: “Are deep-learning approaches really more efficient than traditional approaches?”. The answer, as determined in this experiment seems to be “yes” with regard to generality.

## Limitations and strengths

Previous researchers concerned with evaluating pre-processing tasks considered only different types of stemming and stop words removal [27, 28]. Even in English, a recent study only compared 16 pre-processing techniques [76], here we have reviewed the literature and identified 14 variants of normalizing Arabic letters in addition to 12 pre-processing techniques that have been used for Arabic classification tasks. Future study should focus on investigating the impact of pre-processing on more than one data set. It should also focus on the impact of Arabic-specific normalization.

It should be noted that other newer deep learning techniques, such as autoencoders to learn features, or transformer-based language model such as BERT (Bidirectional Encoder Representations from Transformers [126] and, for Arabic, AraBERT [127]) may well outperform the models used here. However, the focus of this study is to use common deep learning architectures with pre-trained word embeddings and compare them with common traditional machine learning models frequently used in the related literature.

Another limitation is in Phase 3 in the first experiment (Fig. 2). Although we used brute force in combing the pre-processing techniques and carried out 128 experiments for this task, we did not consider the order in which the pre-processing techniques were applied, which might have had an impact on the results. It is also possible that different combinations applied to different traditional approaches may have yielded more significant gains and thus led to other approaches overtaking MNB.

## Conclusion

The goal of this paper was to evaluate the impact of pre-processing techniques on traditional algorithms, and we discovered that most of the techniques did not improve the accuracy of the baseline model. In addition, three out of four pre-processing techniques used in the final model for the first experiment are language specific. For the deep learning methods, we found that the BLSTM architecture performed better than the CNN architecture and the MNB classifier. BLSTM with Mazajak CBOW pre-trained word embedding performed the best on the first data set, while BLSTM with Mazajak Skip-Gram performed the best with unseen data. Overall, it was found that BLSTM with Mazajak Skip-Gram pre-trained word embedding was the best model with an F_1_ score of 89.22% for the first data set and 75.2% for the second data set.

## Data Availability

The data sets are available to public and can be found in: https://tinyurl.com/79uykb5z

## Appendices

### Appendix 1

#### MNB

See Table 9.

**Table 9:** Hyperparameters optimized for NB

*Hyperparameters*	'tfidf__norm'	'clf__alpha'
Range	('l1', 'l2'),	[1, 1e-1, 1e-2]

#### SVM

See Table 10.

**Table 10:** Hyperparameters optimized for SVM

*Hyperparameters*	'clf__C'	'clf__loss'
Range	[.05, .12, .25, .5, 1, 2, 4]	['squared_hinge','hinge']

#### LG

See Table 11.

**Table 11:** Hyperparameters optimized for LG

*Hyperparameters*	'clf__C'	'clf__loss'
Range	[0.001,0.01,0.1,1,10,100]	['l1','l2']

#### KNN

See Table 12.

**Table 12:** Hyperparameters optimized for KNN

*Hyperparameters*	clf__algorithm	clf__n_neighbors	clf__leaf_size
Range	['auto', 'kd_tree']	[2, 4, 6, 8, 9, 10, 11, 12]	[2, 9, 16, 20, 26, 31, 50, 70]

### Appendix 2

File 1: results of the evaluation of the best functions.

### Appendix 3

See Tables 13 and 14.

**Table 13:** Optimal hyperparameters of the CNN

	CNN_Mazajak sg	CNN_Mazajak cbow	CNN_fasttext	CNN_arwordvec sg	CNN_arwordvec cbow	Cnn arvec sg	Cnn arvec cbow
cov_filter	32	32	1	1	32	1	32
cov_filter1	32	32	1	32	32	32	32
cov_filter2	1	32	1	32	32	1	32
cov_kernel	32	32	32	32	32	1	1
cov_kernel1	1	1	32	1	32	32	32
cov_kernel2	1	1	1	1	32	1	1
pool_filter	32	32	1	32	1	1	1
cov1_activation	relu	sigmoid	relu	relu	relu	sigmoid	relu
cov1_activation1	relu	relu	relu	relu	sigmoid	relu	relu
cov1_activation2	sigmoid	relu	relu	relu	relu	relu	relu
dropout_1	0.0	0.0	0.6	0.600	0.600	0.300	0.000
dense_units	380	20	20	20.000	380.000	20.000	380.000
Dense activatino	relu	relu	sigmoid	relu	relu	sigmoid	sigmoid
dropout_2': 0.0	0.0	0.000	0.5	0.0	0.0	0.5	0.0
learning_rate	0.01	0.001	0.001	0.001	0.001	0.001	0.001
Batch size	40	40	40	40	40	40	300

**Table 14:** Optimal hyperparameters of the BLSTM

	Mazajak sg	Mazajak cbow	Fasttext	ArWordVec sg	ArWordVec cbow	arvec sg	AraVec cbow
Neurons	280	360	20	500	500	500	500
Drop rate	0.2	0	0.5	0.3	0.2	0.5	0.2
Drop rate	0.2	0	0	0.2	0	0	0
Learning rate	0.001	0.001	0.01	0.001	0.001	0.01	0.01
Batch size	120	40	40	300	300	40	300

## Abbreviations

SVM: Support vector machine
NB: Naïve Bayes
KNN: K-Nearest Neighbors
TF-IDF: Term Frequency–Inverse Document Frequency
CNN: Convolutional Neural Network
BTO: Binary Term Occurrence
LSTM: Long Short-Term Memory
CBOW: Continuous Bag-of-Words
ML: Machine Learning
BLSTM: Bidirectional Long Short-Term Memory
MNB: Multinomial NB
